# Editorial: DAMPs Across the Tree of Life

**DOI:** 10.3389/fimmu.2021.844315

**Published:** 2022-02-01

**Authors:** Seung-Yong Seong, Polly Matzinger, Walter Gottlieb Land

**Affiliations:** ^1^ Department of Microbiology and Immunology, Seoul National University College of Medicine, Seoul, South Korea; ^2^ Wide River Institute of Immunology, Seoul National University, Seoul, South Korea; ^3^ Ghost Lab., Division of Intramural Research, National Institute of Allergy and Infectious Diseases, National Institutes of Health, Bethesda, MD, United States; ^4^ German Academy for Transplantation Medicine, Munich, Germany; ^5^ Molecular ImmunoRheumatology, INSERM UMR_S1109, Laboratory of Excellence Transplantex, University of Strasbourg, Strasbourg, France

**Keywords:** damage-associated molecular patterns, immunity, inflammation, innate, pattern-recognition

“The *danger/injury model*” of Immunity, formulated twenty seven years ago, holds that the immune system does not care about self vs. nonself, but rather that cell stress/tissue injury are the critical features that initiate an immune response (1, 2). The core of the danger/injury model suggests that injured/stressed cells generate “danger signals”, also known as damage-associated molecular patterns (DAMPs) (3, 4), or “alarmins” (5), and that these DAMPs initiate both healing and immunity. In addition, some DAMPs also drive inflammation-resolving responses (6), the aim always being to restore homeostasis. 

It currently appears that all organisms on our planet use DAMPs to signal that cell stress and tissue injury have occurred, be it of sterile or infectious nature (7, 8). Typical examples of such defensive responses across the tree of life are: in plants, volatile DAMPs promote an indirect defense response against insect herbivores (9, 10); in *Drosophila*, the Toll-mediated antimicrobial defense pathway is triggered by a yet-unidentified DAMP (11); in *C. elegans*, the microbe-induced bloating of the intestinal lumen triggers (still unidentified) danger signals to activate a broad innate immune defense response (12); in shrimps, the innate immune response to infections is presaged by the release of HMGB1 (13); and, in fish/lampreys, L-HMGB1-mediates or initiates pathogen defense (14).

There is also another side to this coin: Emerging evidence from an increasing number of experimental and clinical studies suggests that dysregulated emission of DAMPs plays a critical role in the induction of pathologies and diseases; again, a phenomenon that seems to exist across the tree of life. Thus, excessive production of DAMPs may promote such pathologies/diseases as exemplified in plants by the hypersensitive response (HR), death of individual cells, and reduced growth of the entire organism (15); in corals, by increased heat stress-driven coral bleaching (16); and in humans, by hyperinflammation as a typical feature of COVID-19-associated sepsis (17) and the initiation of autoimmune diseases (in this issue). This unique scenario, which runs throughout the tree of life, is addressed in this Research Topic. In accordance with the tenor of the theme, the topic has been divided into three volumes covering *plants* (Vol. 1), *regulated cell death and immune responses* (Vol. 2), and *human diseases* (Vol. 3).

## Volume 1: Plants

The ten articles presented in Vol. 1 are devoted to the role of DAMPs in plant defenses. The topic is comprehensively introduced by Vega-Muñoz et al. who review the dynamic molecular mechanisms of wound responses in plants upon biotic or abiotic stress conditions. Notably, the plant defense program also includes damage-initiated regeneration processes that are profoundly described by Christiaens et al. within this Research Topic, without expressly referring to the role of DAMPs.

Following are three papers on the role of extracellular self-DNA released from damaged or dying cells, molecules believed to operate as typical DAMPs from early on in evolution (18, 19). Thus, Barbero et al., show that, in tomatoes, extracellular self-DNA fragments, operate as potent DAMPs, triggering plant defense responses typical of biotic responses to pathogens and herbivores, particularly to those that cause intensive plant cell disruption or cell death. Other lines of studies on the model of wilt and root rot of *Capsicum annuum* L. plants performed by Serrano-Jamaica et al. show that the use of fragmented DNA derived from plant phytopathogens (i.e., using DNA as PAMPs) can also promote a defense response, as documented by control of the severity of the wilt of the chili caused by these phytopathogens, reducing mortality by 40%. Monticolo et al. then provide an evolutionary overview on extracellular DNA in its various functions, including its protective role as potent DAMPs derived/released from different sources, such as root extracellular traps, surrounding the plant root cap.

Another class of extracellular DAMPs involved in plant immune defense responses and included in this Research Topic are oligosaccharides derived from the plant cell wall components, homogalacturonan, cellulose, xyloglucan, mannan, and arabinoxylan. Thus, Mélida et al. report on studies in which they identified arabinoxylan-oligosaccharides as a novel group of DAMPs active in plants. The group characterized 33-α-L-arabinofuranosyl-xylotetraose as a highly active structure, triggering strong immune responses in *Arabidopsis thaliana* and enhancing crop disease resistance. In another paper, Barghahn et al., present an analysis, in *Hordeum vulgare* and *Arabidopsis thaliana*, of the eliciting capacity of β-1,3/1,4-glucan oligosaccharides (which are also present in phytopathogens). The investigators found that these DAMPs promote canonical pattern-triggered immunity responses in both monocot and dicot plant species, suggesting a potential dual function as DAMPs (derived from plant cell wall) and/or MAMPs (derived from phytopathogens) in a plant lineage-dependent manner. Pontiggia et al. report on two members of this family of DAMPs (i.e., oligogalacturonides and cellodextrins), showing that oxidation of oligogalacturonides and cellodextrins by specific oxidases not only inactivates their DAMP activity but also makes them a significantly less desirable food source for microbial pathogens. In view of these findings, the authors suggest that oxidation and inactivation of these DAMPs may be a general strategy for plants to control the homeostasis of DAMPs, whereby *“DAMP oxidases can be considered in the broadest sense as DAMP suppressors: i.e., SAMPs that maintain a balanced level of signals in plants”*. Of note, these findings provide the first evidence for the concept that DAMP-promoted initiation of inflammation and SAMP-driven inflammation resolution, aimed at restoring homeostasis upon stress and injury, is a universal, evolutionarily ancient phenomenon.

Another plant DAMP that has been shown to elicit robust innate immune responses upon stress and injury in mammals is extracellular ATP (20). The role of ATP in plants has been indirectly addressed by Kumar et al. by focusing on its receptor, the purinoceptor P2K1/DORN1. The receptor is known to confer plant resistance to foliar biotrophic, hemibiotrophic, and necrotrophic pathogens. In their report, the investigators describe the contribution of P2K1 to resistance in *Arabidopsis* against *Rhizoctonia solani*, a broad host range, necrotrophic, soilborne fungal pathogen. From their observations, the research group hypothesized that extracellular ATP, released upon *R. solani*-induced cellular damage (i.e., necrosis), activates the purinoceptor, which initiates a signaling cascade leading to the induction of defense genes.

Another group of DAMPs thought to be involved in plant defense are the plant-derived volatile organic compounds (VOCs), in particular, herbivory-induced plant VOCs. This highly interesting category of DAMPs that has recently gained much attention is comprehensively reviewed by Meents and Mithöfer within this Research Topic. In their overview, the authors highlight the damage-induced volatiles (denoted “DIVs”) by focusing on their origin, chemical nature, physiochemical properties, biological relevance, and putative function in plant-to-plant communications.

Together, the articles presented in Vol. 1 of this Research Topic impressively support current views about host immune defense responses against stress and injury as a universal phenomenon common to all living species in their daily struggles.

## Volume 2: Regulated Cell Death and Immune Responses

The articles in Vol. 2 are devoted to DAMP-promoted immune responses, Condé et al. studied the correlation between a heat shock insult, transcription of immune response genes, and subsequent susceptibility to *Plasmodium berghei* infection in *Anopheles albimanus*. They observed, among other things, that heat shock, documented by upregulated heat shock proteins (e.g., HSP70), increases the resistance of mosquitoes to Plasmodium invasion (note: heat shock proteins are generally recognized as DAMPs). The authors concluded that the data provided by their experiments *“could help the understanding of infection transmission under the ever more common heat waves”*. Roudaire et al. reviewed reports from the literature, comparing the role of PAMP/DAMP-sensing pattern recognition receptors (PRRs) and downstream signaling in both animal and plant cell death. The authors focused on PRR-mediated signaling pathways promoting induction of subroutines of regulated cell death (RCD, such as apoptosis, necroptosis, and pyroptosis), associated with release of DAMPs to combat microbial attack. Of note, in plants, a special form of regulated necrosis (displaying some feature of necroptosis or pyroptosis) is associated with a PRR-triggered HR [see above, and also (15)]. In their attempt to highlight similarities and specificities of the immune responses existing in mammals and plants, the authors concluded: *“The generic name HR does not reflect the complexity of signaling pathways and, as in mammals, recognition of PAMPs, DAMPs or effectors does probably not lead to the engagement of one unique cell death response but likely activates different cell death-signaling pathways with specific features and outcomes. The cell death signal also likely depends on the cell type and plant species”*.

Another thread of information within this Research Topic is provided by articles dealing with productive sources of DAMPs emissions, that is, RCD and extracellular vesicles (EVs). Thus, Cheng et al. discuss the role of infection-induced immune cell death in sepsis, often associated with multiple organ dysfunction syndrome that is believed to be promoted by release of large amounts of DAMPs. Picca et al. discuss the highly complex, dynamic, and variable intracellular and extracellular trafficking of DAMPs and EVs, focusing on the generation of EV and mitochondrial-derived vesicles along the endocytic pathway, thought to be involved in cancer and neurodegeneration. They argue that studying these mechanisms is needed to reveal relevant pathogenic pathways and novel targets for drug development.

Further evidence for a pathogenetic role of DAMPs in human diseases is provided by Ueno et al., showing that prednisolone suppresses the extracellular release of HMGB1 and associated inflammatory pathways in Kawasaki disease. The authors suggest that prednisolone treatment during the acute phase of the disease may ameliorate HMGB-1-mediated inflammatory responses in Kawasaki disease vasculitis. Also, Wyczanska and Lange-Sperandio review findings from studies on the murine model of unilateral ureteral obstruction, demonstrating that subroutines of RCD and release of various DAMPs promote inflammatory and fibrogenic responses in this disease. The authors conclude that research into DAMPs as biomarkers and their use in therapeutic applications, especially regarding kidney inflammation and fibrosis, is a promising field for future research.

Two articles deal with new insights into activation mechanisms of RCD. Smith covers the innate immune receptor *cGAS–stimulator of interferon genes* (STING) that senses endogenous and exogenous DNA. In fact, emerging evidence suggests that the activation of STING-dependent signaling is also implicated in the process of RCD, such as apoptosis, pyroptosis, and necroptosis (21). Smith’s “curiosity arousing” title heralds a review of evidence in support of the hypothesis that endoplasmic reticulum stress may activate STING in the absence of an obvious ligand *via* calcium/reactive oxygen species-mediated mitochondrial damage and release of mitochondrial DNA (a known DAMP). Evidence collected from various experiments and described in this review, indeed, support this concept of a 3-way communication that is critical in triggering STING-mediated RCD *via* initial intracellular stress and damage. Another review article related to RCD, authored by Xu et al., addresses DAMP/PAMP-promoted molecular mechanisms underlying potassium efflux for NLRP3 inflammasome activation, known to lead to pyroptotic cell death. The authors summarize current knowledge on ion channels and pore-forming proteins, including P2X7 receptor, Gasdermin D, pannexin-1, and two-pore domain potassium channels, showing them to be mainly involved in potassium efflux-dependent activation mechanisms. They propose that these mechanisms present viable therapeutic targets for NLRP3 inflammasome-related diseases.

Simultaneous activation of the inflammatory response and clotting cascade following tissue injury is a phylogenetically ancient survival strategy. In fact, the linkage between inflammation and coagulation can be traced back to early events in eukaryotic evolution, before the separation of plants and invertebrate animals from the vertebrates [for more information, see (22)]. Shi et al. addressed this important emerging issue by reviewing rodent models of immunothrombosis and the evolving evidence that extracellular DNA is a driver of immunothrombosis and discussing challenges and prospects for extracellular DNA as a potential therapeutic target. Another review on immunothrombosis is by Watanabe-Kusunoki et al., who report on the hypercoagulable state after endothelial injury, during which thrombomodulin is released into the intravascular space by proteolytic cleavage of the endothelium component. The authors refer to recent studies revealing that recombinant thrombomodulin functions as an inflammatory regulator beyond hemostasis through various molecular mechanisms, including neutralization of DAMPs like histones and HMGB1, and suppression of excessive activation of the complement system. Given the information in this review, it is tempting to denote thrombomodulin as a SAMP.

## Volume 3: Human Diseases

Eight articles included in Vol. 3 are devoted to the role of DAMPs in human diseases or experimental models related to them; quite admittedly a topic that is still in its infancy. Two papers deal with the phenomenon of ischemia-reperfusion injury (IRI) that is known to aggravate and amplify tissue damage in acute diseases such as myocardial infarction (MI) and stroke, as well as initiate innate/alloimmune processes leading to allograft rejection. In both situations, the ischemic injury induces various subroutines of RCD [e.g., ferroptosis (23)] associated with release of large amounts of DAMPs, which, *via* promotion of inflammatory responses, lead to additional local tissue injury as well as operate as mediators of remote organ injury in sterile inflammation (as shown in other lines of studies on trauma) (24). Here, Silvis et al. present a review on MI and heart transplantation. The authors summarize the current evidence for involvement of DAMPs and PRRs in the inflammatory response after MI and cardiac transplantation and, further discuss various current therapeutic approaches targeting this complex scenario. Furubeppu et al., using a mouse model of skeletal muscle IRI, show that HMGB1, but not histone H3 (also a DAMP), translocate from the nucleus to the cytoplasm during skeletal muscle ischemia, and are released into the systemic circulation immediately after reperfusion. Indeed, as discussed by the authors, this study supports the earlier suggested idea that DAMPs locally released from damaged muscles might contribute to the remote organ injury, such as acute lung injury and acute kidney injury (AKI).

The prominent role of DAMPs in AKI is also documented by two contributions from other biomedical fields. Thus, Masum et al., in studies on a model of obstructed kidneys, show a novel mechanism underlying glomerular lesion development in obstructive nephropathy in young and old mice. The investigators clarified by their experiments that glomerular lesions develop during obstructive nephropathy owing to podocyte injury through the overexpression of TLR8 in podocytes from both obstructed and collateral kidneys of young and old mice. Notably, the researchers observed that the glomerular expression of TLR8 positively correlated with its endogenous ligand *glomerular miR-21*, acting as a DAMP (as do other RNAs). Moreover, elevated levels of serum miR-21 were found to activate TLR8 in the podocytes of the collateral kidney and induce glomerular lesions through podocyte injury. Again, these studies nicely insinuate a possible role of DAMPs in contributing to remote organ damage. Another article on the role of DAMPs in AKI - this time, a review on the role of DAMPs in septic AKI - was presented by Ludes et al. In their paper, the authors highlight a limited number of recognized DAMPs, including kidney-specific DAMPs such as uromodulin (also denoted Tamm-Horsfall protein) and non-kidney-specific DAMPs such as HMGB1, histones, biglycan, and extracellular DNA. They describe the crucial role of these DAMPs in septic AKI as initiating and then amplifying the pathophysiological inflammatory process and cover the most recent insights in AKI recovery.

The spectrum of disorders in which DAMPs have been shown to be pathogenetically implicated comprises autoinflammatory and autoimmune diseases as well. Examples of systemic autoinflammatory diseases are presented by Jung et al., who review the role of interactions between TLRs and their endogenous ligands, i.e., DAMPs, in the pathogenesis of systemic juvenile idiopathic arthritis and adult-onset Still’s disease. The authors report that several DAMPs, such as S100 proteins, HMGB1, and myeloperoxidase-DNA, play a role in the development or severity of these disorders by aggravating inflammation. Finally, they recommend that these DAMPs be used as valuable biomarkers for diagnosis and disease activity in patients with these two diseases. Felux et al. refer to the role of DAMPs in autoimmune diseases by choosing systemic lupus erythematosus (SLE) as a classical example of these disorders. As known from earlier studies, in SLE, the chromatin-shaping extracellular nucleosomes (i.e., DNA + histones) behave as both nuclear *autoantigens* eliciting specific anti-nucleosome autoantibodies and as *DAMPs* promoting innate immune proinflammatory and – *via* activation of DCs – adaptive autoimmune responses. In SLE, impaired clearance of chromatin leads to accumulation of plasma mono- and oligonucleosomes, thereby increasing the concentration of both DAMPs and autoantigens. The reason for impaired chromatin clearance in SLE patients is a decreased activity of DNase1, a major nuclease in body fluids that is thought to play an important role in degrading chromatin. Interestingly, DNase1 is linked - in terms of convergently transcribed genes - to *tumor necrosis factor receptor-associated protein-1* (Trap1), a mitochondrial chaperone that regulates stress responses. Felux et al., as a result of complex studies on DNase1-deficient and DNase1/Trap1-deficient mice not mentioned here, argue that combined low activities of both DNase1 and Trap1 lead to impaired degradation of chromatin *in vitro*, delayed chromatin clearance *in vivo*, accumulation of mono-nucleosomes (in terms of their pathogenetic function as DAMPs), and, therefore, enhanced activation of immune cells. The authors emphasize their findings in the title of their paper in terms of an *argumentum e contrario: “Deoxyribonuclease 1-mediated clearance of circulating chromatin prevents from immune cell activation and proinflammatory cytokine production, a phenomenon amplified by low Trap1 activity: consequences for Systemic Lupus Erythematosus”*.

Two papers of this Research Topic are directly devoted to properties of distinct DAMPs, namely interleukin-33 (IL-33) and extracellular matrix (ECM) components. Perez et al. report on a clinical study aimed at exploring the role of IL-33 in celiac disease. Interleukin-33, when released after injury, stress, or RCD, is recognized as a DAMP and interacts with the surface receptor, ST2. Perez et al., in their studies, found an increased expression of IL-33 in duodenal mucosa of active celiac disease patients. In particular, locally digested IL-33 releases active fragments, which can contribute to expanding the proinflammatory signal. The investigators demonstrate (among other findings) that both ST2 receptor forms are also upregulated in duodenal mucosa of celiac disease patients. This is accompanied by an increased number of CD8+ST2+ T cells and the expression of T-bet in some ST2+ intraepithelial lymphocytes and lamina propria cells. Given their observations, the authors conclude that their findings *“highlight the potential contribution of IL-33 and its fragments to exacerbate the proinflammatory circuit and potentiate the cytotoxic activity of CD8+ T cells in celiac disease pathology”*. Of note, is that IL-33 has been shown to exert a dual (i.e., pathogenic and protective) function, for example, in inflammatory bowel disease (25) and, thus, may act context-dependently either as a DAMP or a SAMP. Also, but related to another human disorder, namely chronic liver disease, McQuitty et al. reviewed the known pro-/anti-inflammatory and fibrogenic properties of ECM proteins with particular reference to the immunomodulatory properties of the ECM in the context of this hepatic disease. Interestingly, the researchers described the pathogenic function of ECM proteins by dividing them into ECM-associated bioactive molecules, including growth factors, cytokines, and chemokines, as well as fragmented ECM proteins. It is these fragmented proteins, including glycoproteins, glycosaminoglycans, and proteoglycans, generated upon remodeling or during injury, which operate as *bona fide* DAMPs. In view of all the data collected from the literature, the authors concluded that ECM-associated molecules and DAMPs, *via* their triggered signaling cascades, represent new targets in potentially halting, reducing, or reversing fibrogenesis and concomitant inflammatory state in chronic liver disease. Finally, Matzinger suggests, in a wide-ranging theoretical piece, that most autoimmune diseases are not caused by defects in self-tolerance mechanisms, but instead by the dysregulated release or detection of DAMPs.

## Conclusion—Outlook

The articles presented within the three volumes of this Research Topic again document that DAMPs are unique molecules that maintain and restore homeostasis upon any cell stress and tissue injury by activating PRR-triggered inflammation-promoting, inflammation-resolving, and fibrogenic defense responses. However, the papers also reflect the disastrous side of these molecules, which, when emitted in excess, transform these originally beneficial innate immune pathways into uncontrolled, dysregulated responses that lead to severe and eventually fatal pathologies and diseases.

The singularity of describing DAMPs within this Research Topic can be seen in the emerging evidence-based awareness that their pleiotropic, protective → pathogenic functions are increasingly detected in all organisms living on this planet. And it is this new view about the presence of a universal DAMP-driven defense system across the tree of life – reflected by the contributions within this Research Topic - that lends further compelling support for the correctness of the *danger/injury model* in Immunology and will therefore trigger further intensive research work in this area.

## Author Contributions

All authors listed have made a substantial, direct and intellectual contribution to the work, and approved it for publication.

## Conflict of Interest

The authors declare that the research was conducted in the absence of any commercial or financial relationships that could be construed as a potential conflict of interest.

## Publisher’s Note

All claims expressed in this article are solely those of the authors and do not necessarily represent those of their affiliated organizations, or those of the publisher, the editors and the reviewers. Any product that may be evaluated in this article, or claim that may be made by its manufacturer, is not guaranteed or endorsed by the publisher.
